# Patient-Reported Outcome Measures in Patients with Thrombotic Thrombocytopenic Purpura: A Systematic Review of the Literature

**DOI:** 10.3390/jcm12155155

**Published:** 2023-08-07

**Authors:** Alexandre Soares Ferreira Junior, Morgana Pinheiro Maux Lessa, Samantha Kaplan, Theresa M. Coles, Deirdra R. Terrell, Oluwatoyosi A. Onwuemene

**Affiliations:** 1 Department of Medicine, Faculdade de Medicina de São José do Rio Preto, São José do Rio Preto 15090-000, São Paulo, Brazil; 2 Medical Center Library & Archives, Duke University Medical Center, Durham, NC 27710, USA; samantha.kaplan@duke.edu; 3 Department of Population Health Sciences, Duke University School of Medicine, Durham, NC 27710, USA; theresa.coles@duke.edu; 4 Department of Biostatistics and Epidemiology, Hudson College of Public Health, University of Oklahoma Health Sciences Center, Oklahoma City, OK 73104, USA; dee-terrell@ouhsc.edu; 5 Division of Hematology, Department of Medicine, Duke University School of Medicine, Durham, NC 27710, USA

**Keywords:** patient reported outcomes, patient reported outcome measures, health-related quality of life, thrombotic thrombocytopenic purpura

## Abstract

Health-related quality of life (HRQoL) impacts of thrombotic thrombocytopenic purpura (TTP) have been captured in clinical studies using patient-reported outcome (PRO) measures (PROMs) that are validated for other diseases. However, the validity evidence to support the use of existing PROMs in patients with TTP is unknown. In a systematic review of the literature, including studies of adults and children with TTP, we assessed the validity evidence for use of PROMs in clinical research and clinical practice, characterized HRQoL, described the integration of PROMs in clinical practice and evaluated PRO scores for patients with TTP compared with reference populations. From an initial 4518 studies, we identified 14 studies using 16 PROMs to assess general HRQoL domains in patients in remission. No identified studies assessed the validity of PROMs for the context of use of TTP and no studies described PROM integration into TTP clinical practice or evaluated PROMs that were specific for patients with TTP. Moreover, PRO scores were worse in patients with TTP compared with reference populations and other chronic conditions. We conclude that, in patients with TTP, PROMs pick up on important patient experiences not captured by clinical outcomes at present. There is, therefore, a need for studies that assess the validity of existing PROMs in patients with TTP to determine if TTP-specific PROMs specific to patients with TTP should be developed.

## 1. Introduction

Thrombotic thrombocytopenic purpura (TTP) is a life-threatening thrombotic disorder with significant impacts on health-related quality of life (HRQoL) [1,2]. While healthcare providers prioritize clinical outcomes, such as platelet counts, lactate dehydrogenase levels, and ADAMTS13 (a disintegrin and metalloproteinase with a thrombospondin type 1 motif, member 13) activity, HRQoL outcomes are most important to patients [3,4,5,6]. Significant HRQoL impacts reported by patients in remission from acute TTP include fatigue, headache, depression, and cognitive impairment [2,4,7,8,9].

HRQoL impacts are best measured using patient reported outcome measures (PROMs). PROMs are self-completed tools that assess one or multiple outcomes from the patient’s perspective. PROMs capture HRQoL impacts by direct patient self-report, without interpretation through the lens of a healthcare provider [10]. PROMs can help assess disease burden and evaluate response to therapy [11,12,13]. PROMs can also help improve the assessment of HRQoL impacts of therapies by patients, clinicians, and researchers. Additionally, if shown to be valid and reliable, PROMs can be used to support FDA approval of candidate therapies in clinical trials [14].

PROMs are increasingly being used in research studies to measure TTP-associated HRQoL impacts. However, it is not known if and how PROMs have been implemented in TTP clinical settings. It is also not known whether PROMs used at present capture TTP-specific HRQoL impacts or how they may change TTP clinical management. Finally, it is also not known what the validity evidence is for using PROMs for the context of use of TTP. Therefore, to define the landscape of PROMs at present and their validity evidence in studies of patients with TTP, we undertook a systematic review of the literature. As an exploratory objective to characterize TTP-related morbidity, we also reported patient-reported outcomes (PROs) scores across studies.

## 2. Materials and Methods

### 2.1. Search Strategy and Selection Criteria

This study was a systematic review reported in concordance with PRISMA guidelines (Preferred Reporting Items for Systematic Reviews and Meta-Analyses) [15], and registered with PROSPERO (Registration CRD42022347498) [16].

An electronic search of the literature was conducted using the databases Medline (PubMed), Embase (Elsevier), Scopus (Elsevier), and CINAHL (EBSCO) from inception to 10 June 2022. On 10 October 2022, an updated and more sensitive search was completed, to which was added ClinicalTrials.gov. Search keywords were the following: thrombotic thrombocytopenic purpura, quality of life, anxiety, memory, cognition, outcome, attention, and PROMs (for the detailed search strategy, see supplemental Appendix SA Table SA1–SA9).

Study inclusion criteria were the following: (1) studies of patients with a TTP clinical diagnosis (regardless of ADAMTS13 activity) and (2) studies that reported on the use of PROMs (utilization, development, and testing of measurement properties). PROMs could include known PROMs previously identified in the literature or any other patient self-administered instrument.

Studies were excluded for the following reasons: (1) the population included patients with hereditary TTP alone; (2) PROMs were not evaluated; or (3) PRO results were not reported. Also excluded were case reports (sample size of one patient), reviews, commentaries, studies in non-human subjects, and studies in languages other than English.

The search strategy included a manual review of published article reference lists. We also searched were unpublished studies using gray literature sources (ClinicalTrials.gov and Embase). References were compiled in Endnote and articles were uploaded into Covidence systematic review software (Veritas Health Innovation, Melbourne, Australia) [17]. After deduplication, all titles and abstracts were screened by two independent reviewers (ASFJ and MPML) to determine their suitability for a full-text review. Full-text articles were reviewed by the same independent reviewers (ASFJ and MPML). Conflicts were resolved through a discussion between the two reviewers or by a binding vote from a third independent reviewer (OAO).

### 2.2. Data Analysis

Study quality was assessed by two independent reviewers (ASFJ and MPML) using the Joanna Briggs Institute Clinical Appraisal Tools checklist for Cross-Sectional, Cohort, and Clinical Trial Studies [18]. Conflicts were resolved through discussions or by a third independent reviewer (OAO).

As an initial template to capture the data, the reviewers used a data abstraction table. Summary data were extracted from published reports and included the following variables: primary author and year of study; study objective; number of patients; time from last TTP episode; mean time of PROM completion; PROM characteristics (names, number of PROMs used, and domains assessed); PROM clinical practice integration strategies; PROM clinical practice integration impacts; and PRO scores for patients with TTP and the reference population.

As an exploratory objective to characterize TTP-related morbidity across studies, PRO scores comparing patients with TTP to normal controls or the general population were reported. This report assumed that the PROMs were appropriate for patients with TTP within the specified contexts of use. Where applicable, the results of statistical analyses performed within each study are noted.

Finally, in a post hoc analysis, we identified important TTP domains from the patient’s perspective that have not yet been assessed by studies evaluating PROMs in the literature.

## 3. Results

### 3.1. Included Studies

**Figure 1:**
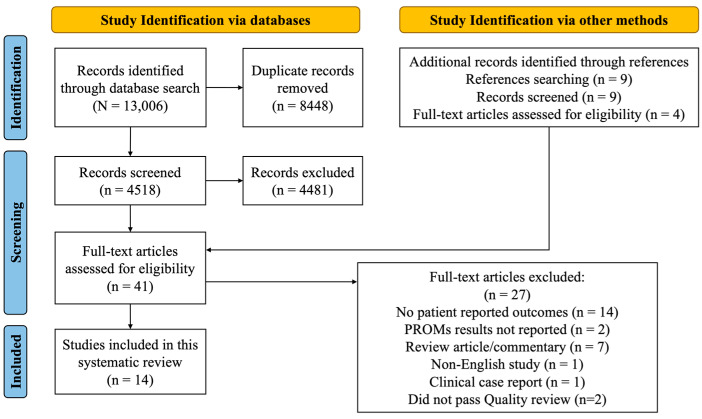
PRISMA flow diagram. There were 13,006 references imported for screening and 8448 duplicates removed. The number of studies screened against title and abstract was 4518. Based on the title and abstract screening, 4481 studies were excluded. Additionally, nine studies were identified manually through references searching. Among those, four studies were assessed for eligibility in the full-text review. Of all full text studies assessed (n = 41), 27 studies were excluded, including 14 with no PROMs, two with no PROMs results, seven review/commentary articles, one clinical case report, and one non-English study. Two studies were also excluded in the quality review. The final number of included studies was 14.

Following deduplication, the search strategy yielded 4518 studies for screening. Nine additional articles were identified through article reference lists. After the abstract review, 41 articles advanced to a full-text review. Of those 41 articles, 25 studies were excluded (see Figure 1). Studies excluded for using instruments that did not meet criteria for PROMs are summarized in Table S1 (Supplementary Materials) [7,13,19,20,21,22,23,24,25,26,27]. Therefore, 16 articles were advanced to a quality review. During the quality review, two additional studies were excluded due to the inability to distinguish the results of patients with TTP from those of other diseases [28,29]. Therefore, the final number of studies for analysis was 14, of which five (36%) were cross-sectional studies, eight (57%) were cohort, and one was a clinical trial (7%). These 14 studies covered 16 PROMs in 970 patients with TTP.

### 3.2. PROMs in Published Studies

Among the 14 studies assessing 16 PROMs (see Table 1 for detailed PROMs data), none reported the use of TTP-specific PROMs. Additionally, none of the studies assessed either strategies for, or the impact of, PROM integration into TTP clinical practice. Only one study evaluated the ease of understanding and relevancy of TTP-specific questions [5]. Additionally, PROMs were mostly assessed for adult patients (TTP typically occurs in adults). Only one study evaluated PROMs in pediatric patients (minimum age: 13 years old); however; the results specific to pediatric patients were not reported [21].

Following recovery from an acute TTP episode, the three most common domains assessed were overall HRQoL, depression, and anxiety. In eight studies (57%), the overall HRQoL was assessed using three different PROMs: Short-Form Health Survey (SF-36) [2,5,13,19,20,66]; Headache Impact Test-6 (HIT-6) [8,13]; and Quality of Life Questionnaire C30 (QLQ-C30) [58]. In eight studies (57%), depression was assessed using six different PROMs: Patient Health Questionnaire Depression Scale 9-item and 8-item instruments (PHQ-9; PHQ-8) [4,21,58,67]; Beck Depression Inventory (BDI-II) [7,52,67]; Inventory of Depressive Symptomatology Self Report (IDS-SR) [9]; Depression, Anxiety and Stress Scale (DASS); [21] and Hospital Anxiety and Depression Scale (HADS) [5,21]. In three studies (21%), anxiety was assessed using three different PROMs: Generalized Anxiety Disorder (GAD-7) [21,58]; Hospital Anxiety and Depression Scale (HADS) [5,21]; and Depression, Anxiety, and Stress Scale (DASS) [21].

Domains less commonly evaluated were post-traumatic stress disorder (PTSD) [52], resilience and life orientation [58], memory [9,58], attention [9,58], executive function [9,58], cognitive function abilities [5], work absenteeism [5], work presenteeism [5], work productivity loss [5], and activity impairment [5] (see Tables S2–S4).

**Figure 2:**
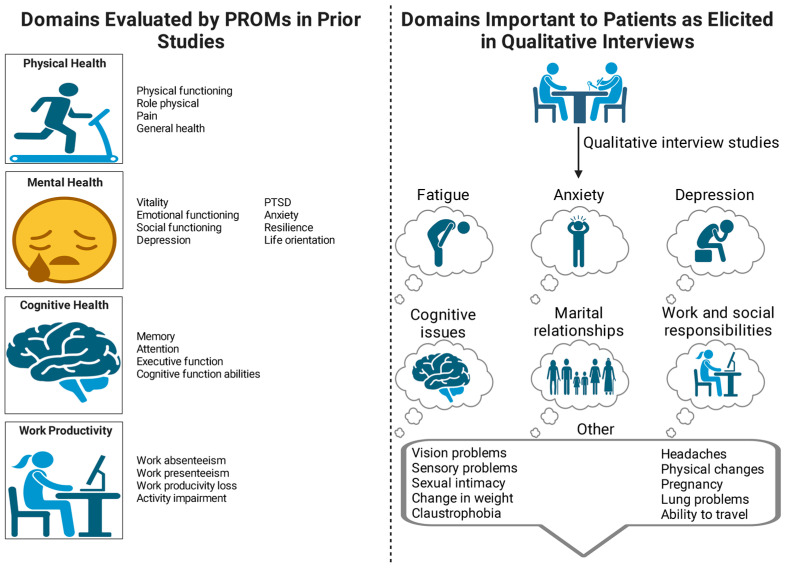
Important domains and impacts from the patient’s perspective. Created with BioRender.com accessed on 12 June 2023.

The post hoc analysis identified four qualitative studies reporting important domains from the patient’s perspective (see Table 2) [5,67,68,69]. These findings are summarized in Figure 2.

### 3.3. PROMs in Unpublished Studies

Three studies were identified through clinicaltrials.gov and the published results were available for one study: the post-HERCULES trial (included in summaries of published studies above). Published results were not available for two studies: the ConNeCT Study (Neurological Complications of TTP), an observational study, and CAPLAVIE (Efficacy of a Personalized Caplacizumab Regimen Based on ADAMTS13 Activity Monitoring in Adult TTP), a clinical trial [26,27]. While the ConNeCT study assessed depression and overall HRQoL using PHQ-9 and SF-36 [26], the CAPLAVIE trial assessed PTSD symptom severity using the PCL-5 [27].

### 3.4. PROMs Capturing the Impact of TTP-Related Morbidity

The results of our exploratory analyses, in which HRQoL domains in patients with TTP were compared with a reference population, are shown in Supplementary Tables S2–S4. In general, following recovery from an acute TTP episode, patients had significant HRQoL impacts.

When PROMs were used to assess overall HRQoL, patients with TTP had worse scores than the general US and Italian population (norms). Additionally, patients with TTP had similar or worse scores than patients with other chronic conditions (anemia, cancer, and depression) [2,5,8,13,19,20,58,66].

Similar findings are shown when PROMs are used to assess specific HRQoL domains. Across the studies, there was a statistically higher prevalence of depression and anxiety in patients with TTP when compared with the control groups [4,7,9,58]. Additionally, in patients with TTP, a positive PTSD screen was prevalent (35%) [52]. Patients with TTP also had worse scores than healthy controls in cognitive function (memory, attention, executive function, and cognitive function abilities) [5,9]. Finally, patients with TTP reported significant impacts on work-related quality of life (see Supplementary Tables S2–S4) [5].

### 3.5. Association between PROMs and TTP Episode Characteristics

Seven studies evaluated the relationship between PROMs and TTP episode characteristics (number of TTP episodes, neurological symptoms, number of therapeutic plasma exchange [TPE] procedures, ADAMTS13 activity during remission, and abnormal magnetic resonance imaging [MRI]). These studies used nine PROMs: SF-36 [2], HIT-6 [8], IDS-SR [9], PHQ-8 or 9 [4,21], HADS [21], DASS [21], GAD-7 [21], BDI-II [7,52], and PCL-5 [52].

Most studies (5/7, 71%) assessed the relationship between TTP episode characteristics and depression and anxiety. In these studies, anxiety and depression were assessed after the TTP episode; however, the time from the TTP episode to administering the PROM was reported by only two studies [7,52]. For these studies, the median time from the TTP episode to administering the PROM was 6.3 and 6.6 years. Depression and anxiety scores were not statistically associated with number of TTP episodes [4,7,9,52], presence of neurological symptoms [4,9,52], number of TPE procedures [4], ADAMTS13 activity during remission [7], and abnormal MRI [21].

One study assessed the relationship between TTP episode characteristics and scores on all SF-36 domains after the initial TTP diagnosis (median time 1.53 years). Scores on all domains were not statistically associated with TTP clinical triggers (idiopathic vs. other), presence of severe ADAMTS13 deficiency, number of TPE procedures, and presence of neurologic symptoms [2].

Additionally, one study assessed the relationship between TTP episode characteristics and HIT-6 scores after the last TTP episode (average time: 3.12 years). Although no statistical analyses were performed, the study suggested that headache severity scores were not associated with the number of TTP episodes, time from last TTP episode, or ADAMTS13 activity level [8].

Finally, one study assessed the relationship between TTP episode characteristics and cognitive deficits using FLei. Cognitive scores were not found to be statistically associated with the number of TTP episodes and the presence of neurological symptoms [9].

## 4. Discussion

Our systematic review identified 14 studies that used 16 PROMs to assess HRQoL domains in TTP. The five main findings were the following: (1) the small number of studies using PROMs in patients with TTP; (2) the absence of studies assessing psychometric properties of PROMs in patients with TTP; (3) the absence of studies evaluating strategies for, and the impact of, integrating PROMs into TTP clinical practice; (4) the absence of PROMs developed specifically for patients with TTP; and (5) decreased HRQoL in patients with TTP when compared with reference populations and other chronic conditions.

PROMs were originally developed for pharmacological research to assess therapeutic effectiveness [71]. More recently, PROMs have been used to support clinical decision making, prioritize patients for surgical procedures, and improve healthcare quality in clinical practice [71,72]. When integrated into clinical practice, PROMs have been shown to optimize provision of patient-centered healthcare, reduce healthcare services utilization, and enhance patient–clinician communication. PROMs have also been shown to increase patient satisfaction and improve HRQoL outcomes [73,74]. In the clinical care of patients with chronic conditions, PROMs have been shown to both improve disease activity and increase survival [73].

Although PROMs are used in hematological research, little is known about their integration into hematology clinical practice [68,75]. For chronic hematological disorders, integrating PROMs into clinical practice may reduce disease burden through the early identification and management of residual symptoms, such as fatigue, depression and anxiety [68,75]. Despite the growing number of PROMs being applied in hematology, integrating PROMs into clinical practice poses some challenges. First, a standard PROM scoring system does not exist and medical providers find it difficult to make clinical decisions using normative-based scores [76]. Second, the integration of PROMs into healthcare systems may be influenced by structural barriers. These barriers include consultation time, absence of implementation recommendations, and prioritization of laboratory outcomes [75]. Third, successful integration relies on using validated PROMs that have undergone psychometric processes to ensure that the PROM measures what is intended [75]. Although PROMs represent the patient’s views of their own health, the outcome assessed may not be important to the patient or to health itself [71]. Therefore, PROMs that are ideal for use in clinical practices are those that both assess important outcomes for patients and providers and are valid, reliable, and specific to the context of use for the disease under study [71].

**Figure 3:**
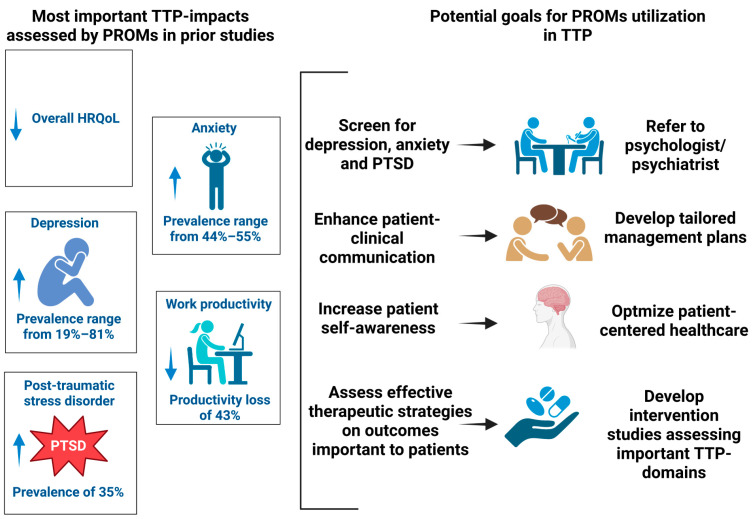
Potential goals for PROM utilization in patients with TTP. Created with BioRender.com accessed on 11 July 2023.

In other chronic conditions with significant impacts on HRQoL, PROMs have been used for clinical purposes [12,77,78,79]. In two prior systematic reviews, PROMs were identified to have the potential to be applied to clinical practice in the following five ways: (1) assess HRQoL in a structured and validated way; (2) foster patient–clinician communication; (3) monitor therapeutic impacts on HRQoL; (4) develop personalized management plans; and (5) increase health awareness [12,77]. Similar clinical applications were also suggested by a qualitative study of 44 patients with TTP in remission. In this study, focus groups (7 groups; n = 25) and individual interviews (n = 19) were conducted to assess TTP residual symptoms and patient–hematologist communication [68]. In all 7 (100%) focus groups and 18 (95%) individual interviews, patients reported residual TTP symptoms that were negatively impacting their activities of daily living [68]. Most patients also reported barriers to communicating these residual symptoms to their hematologists. Based on the abovementioned studies and considering TTP-related morbidity, the potential goals for PROM utilization in TTP are summarized in Figure 3.

Despite these potential applications in TTP care, integrating PROMs into clinical practice poses the aforementioned challenges [75,76]. Therefore, future prospective studies are desired to determine the optimal strategies for integrating PROMs into TTP clinical practice and assess other potential applications.

Our post hoc analysis revealed TTP domains that were felt to be important from the patient’s perspective but were not evaluated in any of the included studies (see Figure 2). In a qualitative study of 50 patients, Holmes et al. identified domains that were important to patients with TTP. These domains included: (1) fatigue; (2) cognitive domains of attention, concentration, and the ability to use language; (3) ability to travel; (4) fear of relapse; and (5) desire/ability to have sex [5]. Another qualitative study of 44 patients with TTP determined that the most important symptoms impacting activities of daily life were cognitive impairment, fatigue, relapse-related anxiety, and depression [68]. Another study by Oladapo et al. reported that domains previously identified as important to patients with hereditary TTP were also relevant to patients with acquired TTP. These domains included vision problems, bruising, dizziness, numbness, sleep disturbance, and fear of relapse [69,70]. Although these domains may be assessed by PROMs available at present, these PROMs cannot be recommended for use in patients with TTP until studies are conducted to evaluate the content validity (understandability and appropriateness) for the context of use of TTP [80]. Thus, content validity studies are needed to facilitate the interpretation of currently available generic PROMs in patients with TTP.

There may be a potential benefit of incorporating PROMs that assess specific domains relevant to patients with TTP [5,68,69,70]. Disease-specific PROMs are designed to capture elements relevant to a specific population or condition and can be used to identify unmet needs and patients priorities [81,82,83]. Rather than disease-specific PROMs, TTP studies have used only generic PROMs. Although widely used across different diseases, generic PROMs may not be sensitive enough to pick up certain specific aspects of the disease under study [81]. Therefore, including disease-specific PROMs in clinical studies would illuminate important information about the impacts of TTP and TTP therapies from the patient’s perspective [5,69,70]. Nevertheless, to guide the development of TTP-specific PROMs, future studies are needed using validated methodological processes. These would include both quantitative and qualitative studies [84].

The strengths of our study lie in its comprehensive review of the landscape of PROMs used in patients with TTP and summary of TTP-related morbidity based on PRO scores. Our review, however, is limited by the overlap of patients across studies, in which PROMs may have been repeatedly administered to the same population. Additionally, since ADAMTS13 activity was not used as an inclusion criterion, patients with other types of thrombotic microangiopathy may have been included among our cohort. Furthermore, since our review included qualitative studies only in a post hoc analysis, some concepts, such as understandability, could not be assessed. Finally, the heterogeneity in domains assessed by different PROMs prevented effective comparisons across studies. Nevertheless, our systematic review is an important milestone in defining the landscape of PROMs in TTP and providing data to guide future studies assessing the use of PROMs in patients with TTP.

## 5. Conclusions

Although PROMs are being used to assess several domains in patients with TTP, studies assessing the psychometric properties of present measures are desired. Additionally desired are qualitative concept elicitation studies. These studies would assess the acceptability of current PROMs for the context of use of patients with TTP. They would also determine whether existing PROMs should be modified for use in patients with TTP or whether there is a need to develop disease-specific PROMs.

## Figures and Tables

**Table 1:** (**a**) PROMs measuring overall health-related quality of life in patients with thrombotic thrombocytopenic purpura *. (**b**) PROMs measuring specific domains of health-related quality of life in patients with thrombotic thrombocytopenic purpura.

(a)		
Measure Average Completion Time	Number of Items Recall Period	Domains Evaluated
SF-36 [5,30] ** <15 min [2]	36 4 weeks	Physical Functioning
		Bodily Pain
		General Health
		Vitality
		Role Physical
		Role Emotional
		Social Functioning
		Mental Health
QLQ-C30 [31,32,33] <9 min [34]	30 1 week	Physical Functioning
		Role Functioning
		Cognitive Functioning
		Emotional Functioning
		Social Functioning
		Global Quality of Life
		Fatigue
		Nausea/Vomiting
		Pain
		Appetite Loss
		Diarrhea
		Dyspnea
		Constipation
		Insomnia
		Financial Impact
HIT-6 [8,35] <2 min [36]	6 4 weeks	Pain
		Role Functioning
		Social Functioning
		Vitality (Energy/Fatigue)
		Cognition
		Emotion Distress
**(b)**		
**Depression and Anxiety Instruments**		
PHQ-8/-9 [12,37] <5 min [37,38,39,40]	8 or 9 *** 2 weeks	Depression
BDI-II [5,41] 5–10 min [42]	21 2 weeks	Depression
GAD-7 [12,43] <2 min [44]	7 2 weeks	Anxiety
HADS [5,45] <5 min [45]	14 1 week	Depression
		Anxiety
IDS-SR [46,47,48] <7 min [49]	30 1 week	Depression
DASS [50] 10 to 20 min [51]	42 1 week	Depression
		Anxiety
		Stress
**Post-traumatic Stress Disorder Instrument**		
PCL-5 [52,53] 5–10 min [54]	20 1 month	PTSD
**Cognitive Function Instruments**		
PROMIS CFAS-SF6a [5,55] Time to complete NR	6 1 week	Cognitive Function Abilities
Flei [9,56,57,58] 10 min [59]	30 6 months	Attention
		Memory
		Executive Functions
**Resilience and Life Orientation Instruments**		
RS-11 [60,61] Time to complete NR	11 N/A	Mental Resistance
LOT-R [58,62] <3 min [63]	10 N/A	Attitude to Life
**Work Activity Instruments**		
WPAI-SHP [64,65] Time to complete NR	6 1 week	Absenteeism
		Presenteeism
		Work Productivity Loss
		Activity Impairment

BDI-II = Beck Depression Inventory II; DASS = Depression, Anxiety, and Stress Scale; FLei = German questionnaire for complaints of cognitive disturbances; GAD-7 = Generalized Anxiety Disorder; HADS = Hospital Anxiety and Depression Scale; HIT-6 = Headache Impact Test-6; IDS-SR = Inventory of Depressive Symptomatology Self Report; LOT-R = Life Orientation Test—Revised; N/A = not applicable; PCL-5 = PTSD checklist for Diagnostic and Statistical Manual of Mental Disorders (DSM-5); Patient-Reported Outcomes Measurement Information System (PROMIS)-CFAS-SF6a = PROMIS Cognitive Function Abilities Subset Short Form 6a; PTSD = Post-Traumatic Stress Disorder; PHQ-8 = Patient Health Questionnaire Depression Scale 8; PHQ-9 = Patient Health Questionnaire Depression Scale 9; QLQ-C30 = Quality of Life Questionnaire C30; RS-11 = Resilience Scale; SF-36 = Short-Form Health Survey; WPAI-SHP = Work Productivity Activity Impairment: Specific Health Problem; * = HIT-6 included among HRQoL instruments to facilitate reading; ** = includes both versions 1 and 2 of the SF-36; *** = PHQ-8 includes only 8 questions.

**Table 2:** Important TTP domains and impacts from the patient’s perspective.

Patient-Reported Domains/Impacts	Prior Studies Assessing Patient’s Perspective				Administered PROMs in Prior Studies
	Oladapo et al. 2018 [69,70]	Holmes et al. 2005 [5]	Terrell et al. 2019 [6]	Kelley et al. 2022 [68]	
Cognitive issues	N/A	X	X	X	FLei [9,58] PROMIS CFAS-SF6a [5]
Fatigue	X	X	X	X	QLQ-C30 [9]
Depression	N/A	X	X	X	PHQ-8/9 [4,6,21,58] BDI-II [6,7,52] IDS-SR [9] HADS [5,21] DASS [21]
Anxiety (including fear of relapse)	X	X	X	X	HADS [5,21] DASS [21] GAD-7 [9,21]
Impact on relationships/family	N/A	X	X	X	SF-36 [2,13,19,20]
Impact on social activities	N/A	X	X	X	SF-36 [2,13,19,20] QLQ-C30 [9]
Impact on work/career	X	X	X	X	WPAI-SHP [5] SF-36 [2,13,19,20]
Experience of flashbacks	N/A	X	N/A	N/A	Not assessed
PTSD	N/A	X	N/A	N/A	PCL-5 [52]
Lack of independence	N/A	X	N/A	N/A	Not assessed
Pain/Headache	X	N/A	N/A	X	HIT-6 [8,13] SF-36 [2,13,19,20] QLQ-C30 [9]
Bruising	X	N/A	N/A	N/A	Not assessed
Sensory problems	X	N/A	N/A	X	Not assessed
Lung problems	N/A	N/A	N/A	X	Not assessed
Claustrophobia	N/A	N/A	N/A	X	Not assessed

BDI-II = Beck Depression Inventory II; DASS = Depression, Anxiety and Stress Scale; FLei = German questionnaire for complaints of cognitive disturbances; GAD-7 = Generalized Anxiety Disorder; HADS = Hospital Anxiety and Depression Scale; HIT-6 = Headache Impact Test-6; IDS-SR = Inventory of Depressive Symptomatology Self Report; N/A = not applicable; PCL-5 = PTSD checklist for Diagnostic and Statistical Manual of Mental Disorders (DSM-5); Patient-Reported Outcomes Measurement Information System (PROMIS)-CFAS-SF6a = PROMIS Cognitive Function Abilities Subset Short Form 6a; PTSD = Post-Traumatic Stress Disorder; PHQ-8 = Patient Health Questionnaire Depression Scale 8; PHQ-9 = Patient Health Questionnaire Depression Scale 9; QLQ-C30 = Quality of Life Questionnaire C30; SF-36 = Short-Form Health Survey; WPAI-SHP = Work Productivity Activity Impairment: Specific Health Problem; X = Assessed.

## Data Availability

Not applicable.

## Supplementary Materials

The following supporting information can be downloaded at: https://www.mdpi.com/article/10.3390/jcm12155155/s1, Appendix SA: Detailed search strategy (Tables SA1–SA9). Table S1: List of instruments excluded in the full-text review; Table S2: patient-reported outcome measures in patients with TTP assessing physical health; Table S3: patient-reported outcome measures in patients with TTP assessing mental health and cognitive function; Table S4: patient-reported outcome measures in patients with TTP assessing work productivity.
