# Supplementary material for: Patient-Reported Outcome Measures in Patients with Thrombotic Thrombocytopenic Purpura: A Systematic Review of the Literature
Source: J Clin Med. 2023 Aug 7;12(15):5155. doi: 10.3390/jcm12155155 (PMC10420299; doi:10.3390/jcm12155155)
Supplement: Supplementary file 1 [file jcm-12-05155-s001.zip › jcm-2479188-supplementary.pdf]

Appendix A – Search Strategy

Appendix SA1. Initial Search on 06/10/2022

Table SA1. Search Strategy – Medline (via PubMed)

Set #	Search Strategy	Results
1 TTP	"Purpura, Thrombotic Thrombocytopenic"[Mesh] OR "Thrombotic Thrombopenic Purpura"[tiab] OR "Thrombotic Thrombocytopenic Purpura"[tiab] OR "Moschcowitz Disease"[tiab] OR "Moschkowitz Disease"[tiab] OR "Schulman-Upshaw Syndrome"[tiab] OR "Schulman Upshaw Syndrome"[tiab] OR "Upshaw-Schulman Syndrome"[tiab] OR "Upshaw Schulman Syndrome"[tiab] OR "Deficiency of Upshaw Factor"[tiab] OR "Congenital Microangiopathic Hemolytic Anemia"[tiab] OR "Familial Thrombotic Microangiopathy"[tiab]	6,431
2 PROMs	"Quality of life"[Mesh] OR "Attention"[Mesh] OR "Anxiety"[Mesh] OR "Memory"[Mesh] OR "Cognition"[Mesh] OR Outcome[tiab] OR outcomes[tiab] OR PROM[tiab] OR PROMs[tiab] OR "quality of life"[tiab] OR Qaly[tiab] OR Qalys[tiab] OR attention[tiab] OR anxiety[tiab] OR attentiveness[tiab] OR anxious[tiab] OR anxiousness[tiab] OR memory[tiab] OR recall[tiab] OR cognition[tiab] OR cognitive[tiab]	3,643,111
3	1 AND 2	717
Validation string	26063190 OR 31558667 OR 31359341 OR 18954401 OR 34238132	5/5

Table SA2. Search Strategy - Embase via Elsevier

Set #	Search Strategy	Results
1 TTP	'thrombotic thrombocytopenic purpura'/d OR 'Thrombotic Thrombopenic Purpura':ti,ab OR 'Thrombotic Thrombocytopenic Purpura':ti,ab OR 'Moschcowitz Disease':ti,ab OR 'Moschkowitz Disease':ti,ab OR 'Schulman-Upshaw Syndrome':ti,ab OR 'Schulman Upshaw Syndrome':ti,ab OR 'Upshaw-Schulman Syndrome':ti,ab OR 'Upshaw Schulman Syndrome':ti,ab OR 'Deficiency of Upshaw Factor':ti,ab OR 'Congenital Microangiopathic Hemolytic Anemia':ti,ab OR 'Familial Thrombotic Microangiopathy':ti,ab	20,167
2 PROMs	'quality of life'/exp OR 'attention'/exp OR 'anxiety'/exp OR 'memory'/exp OR 'cognition'/exp OR Outcome:ti,ab OR outcomes:ti,ab OR PROM:ti,ab OR PROMs:ti,ab OR 'quality of life':ti,ab OR Qaly:ti,ab OR Qalys:ti,ab OR attention:ti,ab OR anxiety:ti,ab OR attentiveness:ti,ab OR anxious:ti,ab OR anxiousness:ti,ab OR memory:ti,ab OR recall:ti,ab OR cognition:ti,ab OR cognitive:ti,ab	6,856,213
3	#1 AND #2	4109
4	#1 AND #2 AND [humans]/lim	3914
5	#4 AND ('article'/it OR 'article in press'/it OR 'conference paper'/it OR 'review'/it)	2213

Table SA3. Search Strategy - Scopus via Elsevier

Set #	Search Strategy	Results
1 TTP	TITLE-ABS-KEY("Thrombotic Thrombopenic Purpura" OR "Thrombotic Thrombocytopenic Purpura" OR "Moschcowitz Disease" OR "Moschkowitz Disease" OR "Schulman-Upshaw Syndrome" OR "Schulman Upshaw Syndrome" OR "Upshaw-Schulman Syndrome" OR "Upshaw Schulman Syndrome" OR "Deficiency of Upshaw Factor" OR "Congenital Microangiopathic Hemolytic Anemia" OR "Familial Thrombotic Microangiopathy")	14,774
2 PROMs	TITLE-ABS-KEY(Outcome OR outcomes OR PROM OR PROMs OR "quality of life" OR Qaly OR Qalys OR attention OR anxiety OR attentiveness OR anxious OR anxiousness OR memory OR recall OR cognition OR cognitive)	7,787,871
3	1 AND 2	3958
4	3 AND (LIMIT-TO (DOCTYPE , "ar") OR LIMIT-TO (DOCTYPE , "cp"))	2736

Table SA4. Search Strategy - CINAHL via EBSCO

Set #	Search Strategy	Results
1 TTP	(MH "Purpura, Thrombotic Thrombocytopenic") OR (TI "Thrombotic Thrombopenic Purpura" OR AB "Thrombotic Thrombopenic Purpura") OR (TI "Thrombotic Thrombocytopenic Purpura" OR AB "Thrombotic Thrombocytopenic Purpura") OR (TI "Moschcowitz Disease" OR AB "Moschcowitz Disease") OR (TI "Moschkowitz Disease" OR AB "Moschkowitz Disease") OR (TI "Schulman-Upshaw Syndrome" OR AB "Schulman-Upshaw Syndrome") OR (TI "Schulman Upshaw Syndrome" OR AB "Schulman Upshaw	1,031

	Syndrome") OR (TI "Upshaw-Schulman Syndrome" OR AB "Upshaw-Schulman Syndrome") OR (TI "Upshaw Schulman Syndrome" OR AB "Upshaw Schulman Syndrome") OR (TI "Deficiency of Upshaw Factor" OR AB "Deficiency of Upshaw Factor") OR (TI "Congenital Microangiopathic Hemolytic Anemia" OR AB "Congenital Microangiopathic Hemolytic Anemia") OR (TI "Familial Thrombotic Microangiopathy" OR AB "Familial Thrombotic Microangiopathy")	
2 PROMs	(MH "Quality of Life+") OR (MH "Attention+") OR (MH "Anxiety+") OR (MH "Memory+") OR (MH "Cognition+") OR (TI Outcome OR AB Outcome) OR (TI outcomes OR AB outcomes) OR (TI PROM OR AB PROM) OR (TI PROMs OR AB PROMs) OR (TI "quality of life" OR AB "quality of life") OR (TI Qaly OR AB Qaly) OR (TI Qalys OR AB Qalys) OR (TI attention OR AB attention) OR (TI anxiety OR AB anxiety) OR (TI attentiveness OR AB attentiveness) OR (TI anxious OR AB anxious) OR (TI anxiousness OR AB anxiousness) OR (TI memory OR AB memory) OR (TI recall OR AB recall) OR (TI cognition OR AB cognition) OR (TI cognitive OR AB cognitive)	1,277,024
3	S1 AND S2	131

Appendix SA2. Updated Search on 10/10/2022

Table SA5. Updated Search Strategy – Medline via PubMed

Set #	Search Strategy	Results
1 TTP	"Purpura, Thrombotic Thrombocytopenic"[Mesh] OR "Thrombotic Thrombopenic Purpura"[tiab] OR "Thrombotic Thrombocytopenic Purpura"[tiab] OR "Moschcowitz Disease"[tiab] OR "Moschkowitz Disease"[tiab] OR "Schulman-Upshaw Syndrome"[tiab] OR "Schulman Upshaw Syndrome"[tiab] OR "Upshaw-Schulman Syndrome"[tiab] OR "Upshaw Schulman Syndrome"[tiab] OR "Deficiency of Upshaw Factor"[tiab] OR "Congenital Microangiopathic Hemolytic Anemia"[tiab] OR "Familial Thrombotic Microangiopathy"[tiab]	6,519
2 PROMs	"Patient Reported Outcome Measures"[Mesh] OR "Quality of life"[Mesh] OR "Attention"[Mesh] OR "Anxiety"[Mesh] OR "Memory"[Mesh] OR "Cognition"[Mesh] OR Outcome[tiab] OR outcomes[tiab] OR PROM[tiab] OR PROMs[tiab] OR "quality of life"[tiab] OR Qaly[tiab] OR Qalys[tiab] OR attention[tiab] OR anxiety[tiab] OR attentiveness[tiab] OR anxious[tiab] OR anxiousness[tiab] OR memory[tiab] OR recall[tiab] OR cognition[tiab] OR cognitive[tiab]	3,752,732
3	1 AND 2	737
Validation string	26063190 OR 31558667 OR 31359341 OR 18954401 OR 34238132	5/5

Table SA6. Updated Search Strategy - Embase via Elsevier

Set #	Search Strategy	Results
1 TTP	'thrombotic thrombocytopenic purpura'/de OR 'Thrombotic Thrombopenic Purpura':ti,ab OR 'Thrombotic Thrombocytopenic Purpura':ti,ab OR 'Moschcowitz Disease':ti,ab OR 'Moschkowitz Disease':ti,ab OR 'Schulman-Upshaw Syndrome':ti,ab OR 'Schulman Upshaw Syndrome':ti,ab OR 'Upshaw-Schulman Syndrome':ti,ab OR 'Upshaw Schulman Syndrome':ti,ab OR 'Deficiency of Upshaw Factor':ti,ab OR 'Congenital Microangiopathic Hemolytic Anemia':ti,ab OR 'Familial Thrombotic Microangiopathy':ti,ab	19953
2 PROMs	'patient-reported outcome'/exp OR 'quality of life'/exp OR 'attention'/exp OR 'anxiety'/exp OR 'memory'/exp OR 'cognition'/exp OR Outcome:ti,ab OR outcomes:ti,ab OR PROM:ti,ab OR PROMs:ti,ab OR 'quality of life':ti,ab OR Qaly:ti,ab OR Qalys:ti,ab OR attention:ti,ab OR anxiety:ti,ab OR attentiveness:ti,ab OR anxious:ti,ab OR anxiousness:ti,ab OR memory:ti,ab OR recall:ti,ab OR cognition:ti,ab OR cognitive:ti,ab	7059780
3	#1 AND #2	4109
4	#1 AND #2 AND [humans]/lim	3912
5	#4 AND ('article'/it OR 'article in press'/it OR 'conference paper'/it OR 'review'/it)	2208

Table SA7. Updated Search Strategy - Scopus via Elsevier

Set #	Search Strategy	Results
1 TTP	TITLE-ABS-KEY("Thrombotic Thrombopenic Purpura" OR "Thrombotic Thrombocytopenic Purpura" OR "Moschcowitz Disease" OR "Moschkowitz Disease" OR "Schulman-Upshaw Syndrome" OR "Schulman Upshaw Syndrome" OR "Upshaw-Schulman Syndrome" OR "Upshaw Schulman Syndrome" OR "Deficiency of Upshaw Factor" OR "Congenital Microangiopathic Hemolytic Anemia" OR "Familial Thrombotic Microangiopathy")	14,987

2 PROMs	TITLE-ABS-KEY(Outcome OR outcomes OR PROM OR PROMs OR "quality of life" OR Qaly OR Qalys OR attention OR anxiety OR attentiveness OR anxious OR anxiousness OR memory OR recall OR cognition OR cognitive)	8019171
3	1 AND 2	4017
4	3 AND (LIMIT-TO (DOCTYPE , "ar") OR LIMIT-TO (DOCTYPE , "cp"))	2736

Table SA8. Updated Search Strategy - CINAHL via EBSCO

Set #	Search Strategy	Results
1 TTP	(MH "Purpura, Thrombotic Thrombocytopenic") OR (TI "Thrombotic Thrombopenic Purpura" OR AB "Thrombotic Thrombopenic Purpura") OR (TI "Thrombotic Thrombocytopenic Purpura" OR AB "Thrombotic Thrombocytopenic Purpura") OR (TI "Moschcowitz Disease" OR AB "Moschcowitz Disease") OR (TI "Moschkowitz Disease" OR AB "Moschkowitz Disease") OR (TI "Schulman-Upshaw Syndrome" OR AB "Schulman-Upshaw Syndrome") OR (TI "Schulman Upshaw Syndrome" OR AB "Schulman Upshaw Syndrome") OR (TI "Upshaw-Schulman Syndrome" OR AB "Upshaw-Schulman Syndrome") OR (TI "Upshaw Schulman Syndrome" OR AB "Upshaw Schulman Syndrome") OR (TI "Deficiency of Upshaw Factor" OR AB "Deficiency of Upshaw Factor") OR (TI "Congenital Microangiopathic Hemolytic Anemia" OR AB "Congenital Microangiopathic Hemolytic Anemia") OR (TI "Familial Thrombotic Microangiopathy" OR AB "Familial Thrombotic Microangiopathy")	1,059
2 PROMs	(MH "Quality of Life+") OR (MH "Attention+") OR (MH "Anxiety+") OR (MH "Memory+") OR (MH "Cognition+") OR (TI Outcome OR AB Outcome) OR (TI outcomes OR AB outcomes) OR (TI PROM OR AB PROM) OR (TI PROMs OR AB PROMs) OR (TI "quality of life" OR AB "quality of life") OR (TI Qaly OR AB Qaly) OR (TI Qalys OR AB Qalys) OR (TI attention OR AB attention) OR (TI anxiety OR AB anxiety) OR (TI attentiveness OR AB attentiveness) OR (TI anxious OR AB anxious) OR (TI anxiousness OR AB anxiousness) OR (TI memory OR AB memory) OR (TI recall OR AB recall) OR (TI cognition OR AB cognition) OR (TI cognitive OR AB cognitive)	1,313,611
3	S1 AND S2	135

Table SA9. Updated Search Strategy – ClinicalTrials.gov

Advanced Search	Condition or Disease: Thrombotic Thrombocytopenic Purpura	118 Studies

Supplementary Tables

Table S1. List of Instruments excluded in the full text review

Study	Instrument
**Published Studies**	
Cataland 2011[1]	CogState test battery
Riva 2020[2]	Digit span
	Digit span backward
	Word Rey List (direct and indirect)
	Trail Making Test A
	Trail Making Test B
	Hamilton Anxiety Rating Scale
	Hamilton Depression Rating Scale
Scully 2022[3]	Repeatable Battery for Assessment of Neuropsychological Status
Han 2015[4]	Montreal Cognitive Assessment
	Repeatable Battery for Assessment of Neuropsychological Status
	Revised Hamilton rating scale for depression
Alwan 2020[5]	National Adult Reading Test
	Wechsler Adult Intelligence Scale-III Verbal or Performance Scale IQ
	Recognition Memory Test (RMT) – Words & Faces
	RMT – Topographical
	Adult Memory & Information Processing Battery
	Doors & People Test
	Graded Naming Test
	Stroop Colour Word Test
	Phonemic Fluency (FAS & Animal)
	Cognitive Estimation Test A
	Modified Card Sort Task
	Hayling Test
	'A' Cancellation Test
	'O' Cancellation Test
	Symbol Digit Modalities Test
Greenberg 1984[6]	Wechsler Adult Intelligence Scale
	Wechsler Memory Scale
	Wide Range Achievement Test
	Benton Visual Retention Test
	Halstead-Reitan Neuropsychological Battery
	Aphasia Screening Test
	Trail Making Tests (Forms A and B)
	Minnesota Multiphasic Personality Inventory
Kennedy 2009[7]	Folstein Mini-Mental State Exam
	Wechsler Adult Intelligence Scale
	Conners Continuous Performance Test, Reaction Time
	Conners Continuous Performance Test, Omission Errors
	Conners Continuous Performance Test, Commission Errors
	Symbol Digit Modalities Test
	Trail Making Test A
	Trail Making Test B
	Grooved Pegboard Test
	Hooper Visual Organization Test
	Phonemic Fluency Test
	Semantic Fluency Test
	California Verbal Learning Test-II, short delay free recall
	California Verbal Learning Test-II, long delay free recall
	California Verbal Learning Test-II, total across trials
	Wechsler Memory Scale-Revised, Logical Memory I
	Wechsler Memory Scale-Revised, Logical Memory II
	Wechsler Memory Scale-Revised, Visual Reproduction I
	Wechsler Memory Scale-Revised, Visual Reproduction II
	Delis-Kaplan Executive Function Battery, Design Fluency subtest
	Wechsler Adult Intelligence Scale-III Matrix Reasoning subtest
**Unpublished Studies From ClinicalTrials.Gov**	
Clinical Outcome Study of ARC1779 Injection in Patients with Thrombotic Microangiopathy[8]	CogState test battery
Study to Assess Efficacy and Safety of Anti-von Willebrand Factor (vWF) Nanobody in Patients with Acquired Thrombotic Thrombocytopenic Purpura (Titan)[9]	Computerized Neuropsychological Test Battery
The ConNeCT Study: Neurological Complications of TTP[10]	Test Your Memory (TYM) test
Efficacy of a Personalized Caplacizumab Regimen Based on ADAMTS13 Activity Monitoring in Adult aTTP (CAPLAVIE)[11]	Mini-Mental State

* ADAMTS13 = A Disintegrin And Metalloproteinase with Thrombospondin Type 1 Repeats 13; TTP = Immune thrombotic thrombocytopenic purpura

Table S2. Patient Reported Outcome Measures in Patients with TTP Assessing Physical Health

Author, Year (N)	PROM	Timing Relative to TTP	Control Group//Norms	Results: Scores	Results: Interpretation
			**Physical Functioning (PF)**		
Lewis 2009[12] 118	SF-36[13,14]	SF-36 was performed with a median interval of 1.53 years (range 0.52-10.29 years) after initial TTP diagnosis	US norms	42.10 vs 50.00 [CI 95% 39.86-44.35]^{M}	TTP vs.US norms<br><br>Patients with TTP had worse score on PF
				NR vs. NR; p=0.018^{M}	Initial Assessment<br>TTP>2years vs. TTP≤2 years<br><br>Patients with TTP with last episode >2years had better score on PF when compared to TTP≤2 years
				NR vs. NR; p>0.05 for all comparisons^{M}	Characteristics of TTP episode<br><br>There was no significant difference in PF when comparing characteristics of the TTP episode
				Slope 1.02; p=0.247	Serial Assessment<br><br>There was no significant improvement in PF scores in a serial assessment over the first 5 years after TTP recovery
Cataland 2011[1] 27	SF-36v2[13,14]	SF-36 was performed with a median interval of 16 months (range 1 - 66 months) after TTP	US norms and control group with anemia, depression and cancer.	40.84 vs 49.59; p=0.0003**	TTP vs. US norms<br><br>Patients with TTP had worse score on PF.
				40.84 vs. 45.35; p=0.0790**	TTP vs. Anemia<br><br>Patients with TTP had similar scores on PF
				40.84 vs 44.14; p=0.1831**	TTP vs. Depression<br><br>Patients with TTP had similar scores on PF
				40.84 vs 40.84; p=0.9994**	TTP vs. Cancer<br><br>Patients with TTP had similar scores on PF
Riva 2020[2] 35	SF-36[13,14]	SF-36 was performed with a median time of 3 years (range 1.4 – 4.5 years) after initial TTP episode	Italian norms	81.40 vs. 84.46; [CI 95% -3.06 (-8.43 to 2.31)]^{M}	TTP vs. Italian norms<br><br>Patients with TTP had similar score on PF
Scully 2022[3] 78	SF-36[13,14]	Sf-36 was performed at baseline and 12, 24 and 36 months after TTP episode	Patients with TTP that received standard care (TPE + steroids and other immunosuppressive agents)	Standard of Care vs. Caplacizumab<br><br>Baseline: 68.3 vs 74.1^{M}<br>Change at 12 months: 1.3 vs 1.5^{M}<br>Change at 24 months: -0.8 vs 5.7^{M}<br>Change at 36 months: 5.7 vs 6.2^{M}	Mean scores were similar between groups and remained stable over time
			**Role Physical (RP)**		
Lewis 2009[12] 118	SF-36[13,14]	SF-36 was performed with a median interval of 1.53 years (range 0.52-10.29 years) after initial TTP diagnosis	US norms	42.04 vs 50.00 [CI 95% 39.85-44.23]^{M}	TTP vs. US norms<br><br>Patients with TTP had worse score on RP
				NR vs. NR; p>0.05 for all comparisons^{M}	Characteristics of TTP episode<br><br>There was no significant difference in RP when comparing characteristics of the TTP episode.
				Slope 1.23; p=0.454	Serial Assessment<br><br>There was no significant improvement in RP scores in a serial assessment over the first 5 years after TTP recovery
Cataland 2011[1]	SF-36v2[13,14]		US norms and control group with anemia, depression and cancer.	36.21 vs 49.78; p<0.0001**	TTP vs. US norms

27		SF-36 was performed with a median interval of 16 months (range 1 - 66 months) after TTP			Patients with TTP had worse score on RP
				36.21 vs. 45.08; p=0.0008**	TTP vs. Anemia<br><br>Patients with TTP had worse score on RP
				36.21 vs. 42.72; p=0.0115**	TTP vs. Depression<br><br>Patients with TTP had worse score on RP
				36.21 vs 40.16; p=0.1522**	TTP vs. Cancer<br><br>Patients with TTP had similar score on RP
Riva 2020[2]<br>35	SF-36[13,14]	SF-36 was performed with a median time of 3 years (range 1.4 – 4.5 years) after initial TTP episode	Italian norms	56.77 vs. 78.21; [CI 95% -21.44 (-26.17 to -16.72)][M]	TTP vs. Italian norms<br><br>Patients with TTP had worse score on RP
Scully 2022[3]<br>78	SF-36[13,14]	Sf-36 was performed at baseline and 12, 24 and 36 months after TTP episode	Patients with TTP that received standard care (TPE + steroids and other immunosuppressive agents)	Standard of Care vs. Caplacizumab<br><br>Baseline: 60.1 vs 65.9[M]<br>Change at 12 months: -5.3 vs 7.7[M]<br>Change at 24 months: 4.1 vs 7.1[M]<br>Change at 36 months: 5.8 vs 3.6[M]	Mean scores were similar between groups and remained stable over time
			**Pain**		
Lewis 2009[12]<br>118	SF-36[13,14]	SF-36 was performed with a median interval of 1.53 years (range 0.52-10.29 years) after initial TTP diagnosis	US norms	45.28 vs 50.00 [CI 95% 42.97-47.60][M]	TTP vs. US norms<br><br>Patients with TTP had worse score on BP
				NR vs. NR; p>0.05 for all comparisons[M]	Characteristics of TTP episode<br><br>There was no significant difference in BP when comparing characteristics of the TTP episode
				Slope -0.09; p=0.941	Serial Assessment<br><br>There was no significant improvement in BP scores in a serial assessment over the first 5 years after TTP recovery
Cataland 2011[1]<br>27	SF-36v2[13,14]	SF-36 was performed with a median interval of 16 months (range 1 - 66 months) after TTP	US norms and control group with anemia, depression and cancer.	44.90 vs 49.88; p=0.0454**	. TTP vs. US norms<br><br>Patients with TTP had worse score on BP
				44.90 vs. 45.46; p=0.8287**	TTP vs. Anemia<br><br>Patients with TTP had similar score on BP
				44.90 vs 42.88; p=0.4254**	TTP vs. Depression<br><br>Patients with TTP had similar score on BP
				44.90 vs 43.74; p=0.6653**	TTP vs. Cancer<br><br>Patients with TTP had similar score on BP
Riva 2020[2]<br>35	SF-36[13,14]	SF-36 was performed with a median time of 3 years (range 1.4 – 4.5 years) after initial TTP episode	Italian norms	62.20 vs. 73.67; [CI 95% -11.47 (-16.39 to -6.55)][M]	TTP vs. Italian norms<br><br>Patients with TTP had worse score on BP
Saultz 2015[15]<br>21	HIT-6[16]	HIT-6 was performed with an average time of 3.12 years after last TTP episode	HIT-6 normative data gathered in 1999 with a total of 1016 general US population that suffered at least one headache in the past 4 weeks	59.9 vs. 51.7; p=0.002[M]	TTP vs. Normative Data<br><br>Patients with TTP had a significantly higher average of HIT-6 scores, suggesting an increased headache severity
				N/A	Characteristics of the TTP episode<br><br>The number of TTP episodes, time from last TTP episode and ADAMTS13 activity seems to not be associated with HIT-6 scores

Scully 2022[3]<br>78	HIT-6[16]	HIT-6 and SF+36was performed at baseline and 12, 24 and 36 months after TTP episode	Patients with TTP that received standard care (TPE + steroids and other immunosuppressive agents)	Standard of Care vs. Caplacizumab<br><br>Baseline: 45 vs 48.2^{M}<br>Change at 12 months: 0.9 vs 0.1^{M}<br>Change at 24 months: 1.2 vs -0.6^{M}<br>Change at 36 months: 0.6 vs 1.4^{M}	Mean scores were similar between groups and remained stable over time
	SF-36[13,14]			Standard of Care vs. Caplacizumab<br><br>Baseline: 68.4 vs 68.0^{M}<br>Change at 12 months: -1.8 vs 0.3^{M}<br>Change at 24 months: -9.4 vs 5.0^{M}<br>Change at 36 months: -5.2 vs 3.7^{M}	
**General Health (GH)**					
Lewis 2009[12]<br>118	SF-36[13,14]	SF-36 was performed with a median interval of 1.53 years (range 0.52-10.29 years) after initial TTP diagnosis	US norms	40.57 vs 50.00 [CI 95% 38.41-42.73]^{M}.	TTP vs. US norms<br><br>Patients with TTP had worse score on GH
				NR vs. NR; p>0.05 for all comparisons^{M}	Characteristics of the TTP episode<br><br>There was no significant difference in GH when comparing characteristics of the TTP episode
				Slope -0.03; p=0.973	Serial Assessment<br><br>There was no significant improvement in GH scores in a serial assessment over the first 5 years after TTP recovery
Cataland 2011[1]<br>27	SF-36v2[13,14]	SF-36 was performed with a median interval of 16 months (range 1 - 66 months) after TTP	US norms and control group with anemia, depression and cancer.	37.69 vs 49.97; p<0.0001**	TTP vs. US norms<br><br>Patients with TTP had worse score on GH
				37.69 vs. 43.82; p=0.0049**	TTP vs. Anemia<br><br>Patients with TTP had worse score on GH
				37.69 vs 40.79; p=0.1372**	TTP vs. Depression<br><br>Patients with TTP had similar score on GH
				37.69 vs 41.73; p=0.0735**	TTP vs. Cancer<br><br>Patients with TTP had similar score on GH
Riva 2020[2]<br>35	SF-36[13,14]	SF-36 was performed with a median time of 3 years (range 1.4 – 4.5 years) after initial TTP episode	Italian norms	59.91 vs. 65.22; [CI 95% -5.31 (-9.38 to -1.24)]^{M}	TTP vs. Italian norms<br><br>Patients with TTP had worse score on GH
Scully 2022[3]<br>78	SF-36[13,14]	Sf-36 was performed at baseline and 12, 24 and 36 months after TTP episode	Patients with TTP that received standard care (TPE + steroids and other immunosuppressive agents)	Standard of Care vs. Caplacizumab<br><br>Baseline: 66.4 vs 53.1 ^{M}<br>Change at 12 months: -.4.0 vs 4.9^{M}<br>Change at 24 months: -1.5 vs 4.8^{M}<br>Change at 36 months: -4.7 vs 3.6^{M}	Mean scores were similar between groups and remained stable over time
**Studies not reporting results by individual domain**					
Falter 2021[17]<br>104	QLQ-C30[18–20]	QLQ-C30 was performed after TTP episode, but median interval not clear	134 randomly selected healthy control group	NR vs. NR; p=0.001 for 2015ME<br>NR vs. NR; p=0.007 for 2016ME	TTP vs. Healthy Control<br><br>Patients with TTP had worse score in all five functional scales as well in the HRQoL in both surveys
				r=-0.754; p<0.0001 for 2015	HRQoL and Depression association

				r=-0.617; p<0.0001 for 2016	The presence of depression was significantly associated with HRQoL in both surveys
Holmes 2021[14] 50	SF-36v2[13,14]	SF-36 was performed after TTP episode, but median interval not clear	US norms	Physical Component: 42.16 vs. 50[M]	TTP vs. US norms<br><br>Patients with TTP were associated with lower scores in physical component
				Physical Component: 40.20 vs. 44.12[M]	TTP≤12months vs TTP>12months<br><br>When compared to TTP episode >12 months, TTP episode ≤12 months was associated with worse mean scores in physical component
Page 2016[21] 34	SF-36	SF-36 was performed after TTP episode and before relapse, with a median time of 1.3 years (range 1.1 to 8.1) in the first assessment and 6.1 years (range 1.1 to 16.4) in the second assessment	US Norms	Physical Component Initial Assessment: 44.4 vs 50; p=0.01[ME]<br><br>Physical Component Final Assessment: 43.6 vs 50; p=0.01[ME]	TTP vs. US norms<br><br>When compared to US norms, patients with TTP performed significantly worse than US norms for both initial and final assessment

BP = Bodily pain; CI = Confidence interval; GH = General health; HIT-6=Headache Impact Test-6; HRQoL = Health related quality of life; N = Number of patients; NR = Not reported; N/A = Not applicable; M = Mean; ME = Median; PF = Physical functioning; QLQ-C30 = Quality of Life Questionnaire-C30; RP = Role physical; SF-36 = Short-Form Health Survey; TPE = Therapeutic plasma exchange; TTP = Thrombotic thrombocytopenic purpura; US = United States; ** = Not clear if scores were reported by mean or median.

Table S3. Patient Reported Outcome Measures in Patients with TTP Assessing Mental Health and Cognitive Function

Author Year (N)	PROMs	Timing Relative to TTP	Control Group//Norms	Results	
				Score	Interpretation
			**Vitality**		
**Lewis 2009**[12] **118**	**SF-36**[13,14]	SF-36 was performed with a median interval of 1.53 years (range 0.52-10.29 years) after initial TTP diagnosis	US norms	43.97 vs 50.00 [CI 95% 41.74-46.19][M]	TTP vs. US norms<br><br>Patients with TTP had worse score on vitality
				NR vs. NR; p=0.028[M]	Initial Assessment<br>TTP>2years vs. TTP≤2 years<br><br>Patients with TTP with last episode >2years had better score on vitality when compared to TTP≤2 years
				NR vs. NR; p>0.05 for all comparisons[M]	Characteristics of the TTP episode<br><br>There was no significant difference in vitality when comparing characteristics of the TTP episode
				Slope 0.37; p=0.681	Serial Assessment<br><br>There was no significant improvement in vitality scores in a serial assessment over the first 5 years after TTP recovery
Cataland 2011[1] 27	SF-36v2[13,14]	SF-36 was performed with a median interval of 16 months (range 1 - 66 months) after TTP	US norms and control group with anemia, depression and cancer.	39.71 vs 49.82; p<0.0001**	TTP vs. US norms<br><br>Patients with TTP had worse score on vitality
				39.71 vs. 43.93; p=0.0533**	TTP vs. Anemia<br><br>Patients with TTP had similar score on vitality
				39.71 vs. 40.13; p=0.8424**	TTP vs. Depression<br><br>Patients with TTP had similar score on vitality
				39.71 vs. 45.84; p=0.0076**	TTP vs. Cancer<br><br>Patients with TTP had worse score on vitality
Riva 2020[2] 35	SF-36[13,14]	SF-36 was performed with a median time of 3 years (range 1.4 – 4.5 years) after initial TTP episode	Italian norms	52.46 vs. 61.89; [CI 95% -9.43 (-13.93 to -4.93)][M]	TTP vs. Italian norms<br><br>Patients with TTP had worse score on vitality
Scully 2022[3] 78	SF-36[13,14]	Sf-36 was performed at baseline and 12, 24 and 36 months after TTP episode	Patients with TTP that received standard care (TPE + steroids	Standard of Care vs. Caplacizumab	

			and other immunosuppressive agents)	Baseline: 50.9 vs 52.4[M]<br>Change at 12 months: -.0.3 vs 2.0[M]<br>Change at 24 months: 5.9 vs 0.6[M]<br>Change at 36 months: 7.5 vs 3.9[M]	Mean scores were similar between groups and remained stable over time
**Emotional Functioning (EF)**					
Lewis 2009[12]<br>118	SF-36[13,14]	SF-36 was performed with a median interval of 1.53 years (range 0.52-10.29 years) after initial TTP diagnosis	US norms	44.00 vs 50.00 [CI 95% 41.43-46.58][M]	TTP vs. US norms<br><br>Patients with TTP had worse score on EF
				NR vs. NR; p>0.05 for all comparisons[M]	Characteristics of the TTP episode<br><br>There was no significant difference in EF when comparing characteristics of the TTP episode
				Slope 1.60; p=0.419	Serial Assessment<br><br>There was no significant improvement in EF scores in a serial assessment over the first 5 years after TTP recovery
Cataland 2011[1]<br>27	SF-36v2[13,14]	SF-36 was performed with a median interval of 16 months (range 1 - 66 months) after TTP	US norms and control group with anemia, depression and cancer.	33.38 vs 49.49; p<0.0001**	TTP vs. US norms<br><br>Patients with TTP had worse score on EF
				33.38 vs. 44.12; p=0.0002**	TTP vs. Anemia<br><br>Patients with TTP had worse score on EF
				33.38 vs. 38.54; p=0.0658**	TTP vs. Depression<br><br>Patients with TTP had similar score on EF
				33.38 vs. 42.47; p=0.0025**	TTP vs. Cancer<br><br>Patients with TTP had worse score on EF
Riva 2020[2]<br>35	SF-36[13,14]	SF-36 was performed with a median time of 3 years (range 1.4 – 4.5 years) after initial TTP episode	Italian norms	42.69 vs. 76.16; [CI 95% -33.47 (-36.86 to -30.08)][M]	TTP vs. Italian norms<br><br>Patients with TTP had worse score on EF
Scully 2022[3]<br>78	SF-36[13,14]	Sf-36 was performed at baseline and 12, 24 and 36 months after TTP episode	Patients with TTP that received standard care (TPE + steroids and other immunosuppressive agents)	Standard of Care vs. Caplacizumab<br><br>Baseline: 75.3 vs 75.0[M]<br>Change at 12 months: -13.2 vs 1.6[M]<br>Change at 24 months: -1.3 vs 0.9[M]<br>Change at 36 months: -4.4 vs -3.7[M]	Mean scores were similar between groups and remained stable over time
**Social Functioning (SF)**					
Lewis 2009[12]<br>118	SF-36[13,14]	SF-36 was performed with a median interval of 1.53 years (range 0.52-10.29 years) after initial TTP diagnosis	US norms	43.75 vs 50.00 [CI 95% 41.24-46.26][M]	TTP vs. US norms<br><br>Patients with TTP had worse score on SF
				NR vs. NR; p=0.004[M]	Initial Assessment<br>TTP>2years vs. TTP≤2 years<br><br>Patients with TTP with last episode >2years had better score on SF when compared to TTP≤2 years
				NR vs. NR; p>0.05 for all comparisons[M]	Characteristics of the TTP episode<br><br>There was no significant difference in SF when comparing characteristics of the TTP episode
				Slope 0.54; p=0.687	Serial Assessment

					There was no significant improvement in SF scores in a serial assessment over the first 5 years after TTP recovery
Cataland 2011[1] 27	SF-36v2[13,14]	SF-36 was performed with a median interval of 16 months (range 1 - 66 months) after TTP	US norms and control group with anemia, depression and cancer.	36.20 vs 49.70; p<0.0001**	TTP vs. US norms.<br><br>Patients with TTP had worse score on SF
				36.20 vs. 43.90; p=0.0044**	TTP vs. Anemia<br><br>Patients with TTP had worse score SF
				36.20 vs. 38.42; p=0.3969**	TTP vs. Depression<br><br>Patients with TTP had similar score SF
				36.20 vs. 41.84; p=0.0455**	TTP vs. Cancer<br><br>Patients with TTP had worse score SF
Riva 2020[2] 35	SF-36[13,14]	SF-36 was performed with a median time of 3 years (range 1.4 – 4.5 years) after initial TTP episode	Italian norms	54.26 vs. 77.43; [CI 95% -23.17 (-26.28 to -20.06)]^{M}	TTP vs. Italian norms<br><br>Patients with TTP had worse score on SF
Scully 2022[3] 78	SF-36[13,14]	Sf-36 was performed at baseline and 12, 24 and 36 months after TTP episode	Patients with TTP that received standard care (TPE + steroids and other immunosuppressive agents)	Standard of Care vs. Caplacizumab<br><br>Baseline: 74.6 vs 74.0^{M}<br>Change at 12 months: -10.6 vs 1.6^{M}<br>Change at 24 months: 0.0 vs -0.8^{M}<br>Change at 36 months: 0.8 vs -2.6^{M}	Mean scores were similar between groups and remained stable over time
			**Depression**		
Deford 2013[22] 37	PHQ-8[23–25]	PHQ-8 was performed with a median interval of 6.3 years (range 1.8 to 16.2 years) after TTP	Control group was from the NHANES	19% vs. 6%; p=0.005	TTP vs. NHANES<br><br>Patients with TTP had a higher major depression prevalence
				NR	Characteristics of the TTP episode (neurological symptoms, TTP relapse and number of TPE procedures)<br><br>The presence of major depression was not associated with the characteristics of the TTP episode
Han 2015[4] 52	BDI-II[26]	BDI-II was performed after TTP episode, but median interval not clear (performed for over 11 years)	Control group was obtained from a survey of 333 primary care medical outpatients	NR. vs.NR; p<0.05	TTP vs. Control group<br><br>Patients with TTP had higher depression rates.
				N/A	Screening for depression<br><br>31 (59%) patients with TTP had a positive screening for depression and 15 (29%) had severe depression at least once
				NR vs. NR; p=0.2919	Depression vs. Age<br><br>Depression was not associated with age
				NR vs. NR; p=0.1424.	Depression vs. Race<br><br>Depression was not associated with race
				NR vs. NR; p=0.7447	Depression vs. Sex<br><br>Depression was not associated with Sex.
				NR vs. NR; p=0.0956	Depression vs. Education<br><br>Depression was not associated with education
				NR vs. NR; p=0.9368	Depression vs. TTP relapse

					Depression was not associated with TTP relapse
				NR vs. NR; p=0.9069	Depression vs. ADAMTS13 activity<10%<br><br>Depression was not associated with ADAMTS13 activity
				r=0.31; p=0.0920	Depression vs. Cognitive Impairment<br><br>Depression was not associated with cognitive impairment
				13.8 vs 14.2; p=08315[M]	Serial Assessments<br><br>There was no significant improvement in BDI-II scores in a serial assessment in patients with TTP who were evaluated in both 2006 and 2014
Chaturvedi 2017[27]<br>209	BDI-II[26]	BDI-II was performed with a median time of 6.66 years after TTP	None	N/A	Depression Prevalence<br><br>The prevalence of depression on patients with TTP was 80.8%.
				[OR 3.65 (95% CI 1.26 – 10.57); p=0.017]	Depression vs. history of depression<br><br>Depression was associated with a history of depression
				[OR 5.86 (95% CI 1.26 – 27.09); p=0.024]	Depression vs. Unemployment due to TTP<br><br>Depression was associated with being unemployed due to TTP
				NR for all	Depression vs. Demographics and Characteristics of TTP episode<br><br>Depression was not significant associated with age, sex, educational level, the presence of neurological manifestation during acute TTP and relapses of TTP
Terrell 2019[28]<br>50	BDI-II[26]<br>PHQ-9[29]	BDI-II and PHQ-9 were performed after TTP episode, but median interval not clear	None	N/A	Depression Prevalence<br><br>The prevalence of moderate or major depression was 48%
Falter 2017[30]<br>104*	IDS-SR[31,32]	IDS-SR was performed after TTP episode, but median interval not clear	A cohort of healthy adults.	68% vs. 13.5%, p<0.001	TTP vs. Healthy adults<br><br>Patients with TTP had a higher depression prevalence
				First survey: r_s=0.157; p value NR<br>Second survey: r_s=0.023; p value NR	Depression vs. Number of TTP episodes<br><br>There was no significant association between the number of the TTP episodes and depression
				First survey: p=0.466<br>Second survey: p=0.367	Depression vs. Neurological Symptoms<br><br>There was no significant association between the presence of neurological symptoms during the TTP episode and depression
				First survey: r_s=0.643; p<0.001<br>Second survey: r_s=0.779; p<0.001	Depression vs. Mental Performance<br><br>Impairment of mental performance correlated with the severity of depression
Alwan 2020[5]<br>29†	PHQ-9[29]<br>HADS[14,33]	PHQ-9, HADS and DASS were performed after TTP episode, but median interval not clear	None	N/A	Depression Prevalence<br><br>The prevalence of depression was 65%
				p=0.15	Depression vs. Abnormal MRI<br><br>There was no relationship between abnormal MRI and the presence of depression

	DASS[34]			58% vs. 8%, p=0.003	Depression vs. Type of TTP<br><br>Depression was more common in patients with immune vs. hereditary TTP.
Falter 2021[17]<br>104	PHQ-9[29]	PHQ-9 was performed after TTP episode, but median interval not clear	134 randomly selected healthy control group	2015: 61.4% vs. 33.8%; p<0.001<br>2016 63% vs. 33.8%; p<0.0001	TTP vs. Healthy Controls<br><br>Patients with TTP had a significantly higher prevalence of depression in both surveys
				2015: r_s=-0.5346; p<0.0001<br>2016: r_s=-0.6447; p<0.0001	Depression vs. Resilience<br><br>The degree of depression was negatively associated with resilience
				2015 Comorbidities; r=0.286; p=0.006<br>2016 Comorbidities; r=0.402; p<0.0001	Depression vs. Comorbidities<br><br>Depression was associated with the presence of other comorbidities
				2015 Sex: p=0.9585<br>2016 Sex: p=0.7514	Depression vs. Sex<br><br>Depression was not associated with sex
				2015 Age: r=-0.119; p=0.2869<br>2016: Age r=-0119; p=0.3140	Depression vs. Age<br><br>Depression was not associated with age
				2015 Physical activity: r=-0.148; p=0.102<br>2016 Physical activity: r=-0.159; p=0.091	Depression vs. Physical Activity<br><br>Depression was not associated with physical activity
				2015Partneship status: r=-0.082; p=0.241<br>2016 Partnership status: r=-0.167; p=0.080	Depression vs. Partnership status<br><br>Depression was not associated with partnership status
Holmes 2021[14]<br>50	HADS[14,33]	HADS was performed after TTP episode, but median interval not clear	None	N/A	Depression Severity<br><br>Patients with TTP had a mean score of 11.78, indicating moderate levels of depression
**Post-Traumatic Stress Disorder (PTSD)**					
Chaturvedi 2017[27]<br>231	PTSD checklist for Diagnostic and Statistical Manual of Mental Disorders (DSM-5) (PCL-5)[35]	PCL-5 was performed with a median time of 6.66 years after TTP	None	N/A	PTSD Prevalence<br><br>The prevalence of a positive screening for PTSD was 35.1%
				[OR 0.96 (95% CI 0.93-0.99]; p=0.017]	PTSD vs. Age<br><br>PTSD was associated with younger age
				[OR 3.57 (95% CI 1.76-7.25); p<0.001]	PTSD vs. Previous dx of anxiety disorders<br><br>PTSD was associated with pre-existing dx of anxiety disorder
				[OR 6.42 (95% CI 2.75-415); p<0.001]	PTSD vs. Unemployment due to TTP<br><br>PTSD was associated with being unemployed due to TTP
				NR	PTSD vs. Demographics and Characteristics of TTP episode<br><br>PTSD was not significant associated with sex, educational level, the presence of neurological manifestation during acute TTP and relapses of TTP
**Anxiety**					
Alwan 2020[5]<br>29	HADS[14,33]	HADS, GAD-7 and DASS were performed after TTP episode, but median interval not clear	None	N/A	Anxiety Prevalence<br><br>The prevalence of anxiety was 55%
	DASS[34]			p=0.48	Anxiety vs. Abnormal MRI

					There was no relationship between abnormal MRI and the presence of anxiety
	GAD-7[36]			48% vs. 8%, p=0.015	Anxiety vs. Type of TTP<br><br>Anxiety was more common in patients with immune than hereditary TTP
Falter 2021[17]<br>104	GAD-7[36]	GAD-7 was performed after TTP episode, but median interval not clear	134 randomly selected healthy control group	2015: 43.7% vs. 28%; p<0.035<br>2016: 53.7% vs. 28%; p<0.008	TTP vs. Healthy Control<br><br>Patients with TTP had a significantly higher prevalence of anxiety in both surveys
Holmes 2021[14]<br>50	HADS[14,33]	HADS was performed after TTP episode, but median interval not clear	None	N/A	Anxiety Severity<br><br>Patients with TTP had a mean score of 12.18, indicating moderate levels of anxiety
**Resilience and Life Orientation**					
Falter 2021[17]<br>104	RS-11[37]	RS-11 and LOT-R were performed after TTP episode, but median interval not clear	134 randomly selected healthy control group	2015: 60 vs. 64; p<0.04[ME]<br>2016: 55 vs. 64; p<0.0001[ME]	TTP vs. Healthy Control (Resilience)<br><br>Patients with TTP had a significantly lower resilience in both surveys
	LOT-R[38]			2015: NR vs. NR; p=0.011[ME]<br>2016: NR vs. NR; p=0.006[ME]	TTP vs. Healthy Control (Optimism)<br><br>Patients with TTP had a significantly lower optimism score in both surveys
				2015: NR vs. NR; p=0.49[ME]<br>2016: NR vs. NR; p=0.63[ME]	TTP vs. Healthy Control (Pessimism)<br><br>Patients with TTP had similar scores in pessimism score in both surveys
**Mental Health in General (MH)**					
Lewis 2009[12]<br>118	SF-36[13,14]	SF-36 was performed with a median interval of 1.53 years (range 0.52-10.29 years) after initial TTP diagnosis	US norms	45.12 vs 50.00 [CI 95% 42.57-47.66][M]	TTP vs. US norms<br><br>Patients with TTP had worse score on MH
				NR vs. NR; p>0.05 for all comparisons[M]	Characteristics of the TTP episode<br><br>There was no significant difference in MH when comparing characteristics of the TTP episode
				Slope -0.29; p=0.723	Serial Assessment<br><br>There was no significant improvement in MH scores in a serial assessment over the first 5 years after TTP recovery
Cataland 2011[1]<br>27	SF-36v2[13,14]	SF-36 was performed with a median interval of 16 months (range 1 - 66 months) after TTP	US norms and control group with anemia, depression and cancer.	38.06 vs 49.61; p<0.0001**	TTP vs. US norms<br><br>Patients with TTP had worse score on MH
				38.06 vs. 45.52; p=0.0018**	TTP vs. Anemia<br><br>Patients with TTP had worse score on MH
				38.06 vs. 36.49; p=0.4965**	TTP vs. Depression<br><br>Patients with TTP had similar score on MH
				38.06 vs. 47.37; p=0.0002**	TTP vs. Cancer<br><br>Patients with TTP had worse score on MH
Riva 2020[2]<br>35	SF-36[13,14]	SF-36 was performed with a median time of 3 years (range 1.4 – 4.5 years) after initial TTP episode	Italian norms	52.46 vs. 66.59; [CI 95% -14.13 (-16.20 to -12.06)][M]	TTP vs. Italian norms<br><br>Patients with TTP had worse score on MH.
Scully 2022[3]<br>78	SF-36[13,14]	Sf-36 was performed at baseline and 12, 24 and 36 months after TTP episode	Patients with TTP that received standard care (TPE + steroids and other immunosuppressive agents)	Standard of Care vs. Caplacizumab	

				Baseline: 72.1 vs 70.1[M]<br>Change at 12 months: -9.3 vs -2.5[M]<br>Change at 24 months: -3.3 vs -2.0[M]<br>Change at 36 months: -0.7 vs -1.5[M]	Mean scores were similar between groups and remained stable over time
**Memory**					
Falter 2017[30]<br>104*	FLei[39]	FLei was performed after TTP episode, but median interval not clear	A cohort of healthy adults. Additionally, the authors used a cohort of healthy controls and depressive patients from the literature.	13.85 vs 7.2[M]<br>13.85 vs. 10.8[M]	TTP vs. Cohort of Healthy Adult<br>TTP vs. Healthy Control from the Literature<br><br>Patients with TTP had lower memory performance
				13.85 vs. 18.3; $p<0.001$[M]	TTP vs. Depressive Cohort from the Literature<br><br>Patients with TTP had a better memory performance
**Attention**					
Falter 2017[30]<br>104*	FLei[39]	FLei was performed after TTP episode, but median interval not clear	A cohort of healthy adults. Additionally, the authors used a cohort of healthy controls and depressive patients from the literature.	13.2 vs 4.8[M]<br>13.2 vs. 9.7[M]	TTP vs. Cohort of Healthy Adult<br>TTP vs. Healthy Control from the Literature<br><br>Patients with TTP had lower attention performance
				13.2 vs. 19.2; $p<0.001$[M]	TTP vs. Depressive Cohort from the Literature<br><br>Patients with TTP had a better attention performance
**Executive Function**					
Falter 2017[30]<br>104*	FLei[39]	FLei was performed after TTP episode, but median interval not clear	A cohort of healthy adults. Additionally, the authors used a cohort of healthy controls and depressive patients from the literature.	11.15 vs 4.7[M]<br>11.15 vs. 8.7[M]	TTP vs. Cohort of Healthy Adult<br>TTP vs. Healthy Control from the Literature<br><br>Patients with TTP had lower executive function performance
				11.15 vs. 19.0; $p<0.001$[M]	TTP vs. Depressive Cohort from the Literature<br><br>Patients with TTP had a better executive function performance
**Cognitive Function Abilities**					
Holmes 2021[14]<br>50	PROMIS CFAS – SF6a[14,40]	PROMIS was performed after TTP episode, but median interval not clear	US norms	39.69 vs. 50[M]	TTP vs. US norms<br><br>Patients with TTP had a lower mean score on cognitive function
**Studies not reporting results by individual domain**					
Holmes 2021[14]<br>50	SF-36v2[13,14]	SF-36 was performed after TTP episode, but median interval not clear	US norms	Mental component: 33.61 vs. 50[M]	TTP vs. US norms<br><br>Patients with TTP were associated with lower scores in mental component
				Mental component: mean 30.59 vs. 36.64[M]	TTP≤12months vs TTP>12months<br><br>When compared to TTP episode >12 months, TTP episode ≤12 months was associated with worse mean scores in mental component
Page 2016[21]<br>34	SF-36	SF-36 was performed after TTP episode and before relapse, with a median time of 1.3 years (range 1.1 to 8.1) in the first assessment and 6.1 years (range 1.1 to 16.4) in the second assessment	US Norms	Mental Component Initial Assessment: 50.3 vs 50; $p = 0.48$[ME]<br><br>Mental Component Final Assessment: 42.9 vs 50; $p = 0.01$[ME]	TTP vs. US norms<br><br>When compared to US norms, patients with TTP performed significantly worse than US norms only in the final assessment
Falter 2021[17]<br>104	FLei[39]	FLei was performed after TTP episode, but median interval not clear	134 randomly selected healthy control group	2015: 28 vs. 22, $p=0.008$[ME]<br>2016: 34 vs. 22, $p<0.0001$[ME]	TTP vs. Healthy Control

					Patients with TTP had worse cognitive performance in both surveys
Falter 2017[30] 104*	FLei[39]	FLei was performed after TTP episode, but median interval not clear	A cohort of healthy adults. Additionally, the authors used a cohort of healthy controls and depressive patients from the literature.	First survey: r_s=0.115; p value NR<br>Second survey: r_s=0.092; p value NR	Cognitive Deficits vs. Number of TTP episodes<br><br>There was no significant association between the number of the TTP episodes and cognitive deficits
				First survey: p=0.193<br>Second survey: p=0.793	Cognitive Deficits vs. Neurological Symptoms<br><br>There was no significant association between the presence of neurological symptoms during the TTP episode and cognitive deficits

BDI-II = Beck Depression Inventory II; CI = Confidence Interval; DASS = Depression, Anxiety and Stress Scale; Dx = diagnosis; EF = Emotional Functioning; FLei = German questionnaire for complaints of cognitive disturbances; GAD-7 = Generalized Anxiety Disorder; HADS = Hospital Anxiety and Depression Scale; Hx = history; IDS-SR = Inventory of Depressive Symptomatology Self Report; LOT-R = Life Orientation Test-Revised; N = number of patients; NR = Not reported; N/A = Not Applicable; NHANES = National Health and Nutrition Examination Survey; M = Mean; ME = Median; MH = Mental Health; MRI = Magnetic resonance imaging; OR = Odds ratio; PCL-5 = PTSD checklist for Diagnostic and Statistical Manual of Mental Disorders (DSM-5); PTSD = post-traumatic stress disorder; PHQ-8 = Patient Health Questionnaire Depression Scale 8; PHQ-9 = Patient Health Questionnaire Depression Scale 9; PROMIS CFAS–SF6a = PROMIS Cognitive Function Abilities Subset Short Form 6a;RS-11 = Resilience Scale; SF = Social Functioning; SF-36 = Short-Form Health Survey; TPE = Therapeutic Plasma Exchange; TTP = Thrombotic Thrombocytopenic Purpura; US = United States; * = included one patient with hereditary TTP; ** = Not clear if scores were reported by mean or median; † = included patients with hereditary TTP.

Table S4. Patient Reported Outcome Measures in Patients with TTP Assessing Work Productivity.

Author, Year (N)	PROMs	Timing Relative to TTP	Control Group//Norms	Results	
				**Scores**	**Interpretation**
**Work Absenteeism**					
**Holmes 2021**[14] **27**	**WPAI-SHP**[14,41]	WPAI-SHP was performed after TTP episode, but median interval not clear	None	N/A	Work Absenteeism<br><br>Among patients with TTP employed, percent work time missed due to TTP was 0%
**Work Presenteeism**					
Holmes 2021[14] 29	WPAI-SHP[14,41]	WPAI-SHP was performed after TTP episode, but median interval not clear	None	N/A	Work Presenteeism<br><br>Among patients with TTP employed, impairment at work due to TTP was reported as 10%.
**Work Productivity Loss**					
Holmes 2021[14] 27	WPAI-SHP[14,41]	WPAI-SHP was performed after TTP episode, but median interval not clear	None	N/A	Work Productivity Loss<br><br>Among patients with TTP employed, work productivity loss reported was 42.73%.
**Activity Impairment**					
Holmes 2021[14] 50	WPAI-SHP[14,41]	WPAI-SHP was performed after TTP episode, but median interval not clear	None	N/A	Activity Impairment<br><br>Among patients with TTP, general activity impairment due to TTP was reported as 70%.

N = number of patients; N/A = Not applicable; TTP = Thrombotic Thrombocytopenic Purpura; WPAI-SHP = Work Productivity Activity Impairment: Specific Health Problem.

REFERENCES

[1] Cataland SR, Scully MA, Paskavitz J, Maruff P, Witkoff L, Jin M, et al. Evidence of persistent neurologic injury following thrombotic thrombocytopenic purpura. Am J Hematol 2011;86:87–9. https://doi.org/10.1002/ajh.21881.

[2] Riva S, Mancini I, Maino A, Ferrari B, Artoni A, Agosti P, et al. Long-term neuropsychological sequelae, emotional wellbeing and quality of life in patients with acquired thrombotic thrombocytopenic purpura. Haematologica 2020;105:1957–62. https://doi.org/10.3324/haematol.2019.226423.

[3] Scully M, de la Rubia J, Pavenski K, Metjian A, Knöbl P, Peyvandi F, et al. Long-term follow-up of patients treated with caplacizumab and safety and efficacy of repeat caplacizumab use: Post-HERCULES study. J of Thrombosis Haemost 2022:jth.15892. https://doi.org/10.1111/jth.15892.

[4] Han B, Page EE, Stewart LM, Deford CC, Scott JG, Schwartz LH, et al. Depression and cognitive impairment following recovery from thrombotic thrombocytopenic purpura: Depression and Cognitive Impairment Following TTP. Am J Hematol 2015;90:709–14. https://doi.org/10.1002/ajh.24060.

[5] Alwan F, Mahdi D, Tayabali S, Cipolotti L, Lakey G, Hyare H, et al. Cerebral MRI findings predict the risk of cognitive impairment in thrombotic thrombocytopenic purpura. Br J Haematol 2020;191:868–74. https://doi.org/10.1111/bjh.17126.

[6] Greenberg DB, Carey RW. The cost of surviving thrombotic thrombocytopenic purpura: case report. J Clin Psychiatry 1984;45:477–9.

[7] Kennedy AS, Lewis QF, Scott JG, Kremer Hovinga JA, Lämmle B, Terrell DR, et al. Cognitive deficits after recovery from thrombotic thrombocytopenic purpura. Transfusion 2009;49:1092–101. https://doi.org/10.1111/j.1537-2995.2009.02101.x.

[8] Clinical Outcome Study of ARC1779 Injection in Patients With Thrombotic Microangiopathy. Available at: https://clinicaltrials.gov/ct2/show/study/NCT00726544. Accessed November, 12, 2022. n.d.

[9] Study to Assess Efficacy and Safety of Anti-von Willebrand Factor (vWF) Nanobody in Patients With Acquired Thrombotic Thrombocytopenic Purpura (aTTP) (TITAN). Available at: https://clinicaltrials.gov/ct2/show/NCT01151423. Accessed November 11, 2022 n.d.

[10] The ConNeCT Study: Neurological Complications of TTP. Available at: https://clinicaltrials.gov/ct2/show/NCT04981028. Accessed September 09, 2022. n.d.

[11] Efficacy of a Personalized Caplacizumab Regimen Based on ADAMTS13 Activity Monitoring in Adult aTTP (CAPLAVIE) Available at: https://clinicaltrials.gov/ct2/show/NCT04720261. Accessed September 09, 2022. n.d.

[12] Lewis QF, Lanneau MS, Mathias SD, Terrell DR, Vesely SK, George JN. Long-term deficits in health-related quality of life after recovery from thrombotic thrombocytopenic purpura. Transfusion 2009;49:118–24. https://doi.org/10.1111/j.1537-2995.2008.01938.x.

[13] Lins L, Carvalho FM. SF-36 total score as a single measure of health-related quality of life: Scoping review. SAGE Open Medicine 2016;4:205031211667172. https://doi.org/10.1177/2050312116671725.

[14] Holmes S, Podger L, Bottomley C, Rzepa E, Bailey KMA, Chandler F. Survival after acute episodes of immune-mediated thrombotic thrombocytopenic purpura (iTTP) – cognitive functioning and health-related quality of life impact: a descriptive cross-sectional survey of adults living with iTTP in the United Kingdom. Hematology 2021;26:465–72. https://doi.org/10.1080/16078454.2021.1945236.

[15] Saultz JN, Wu HM, Cataland S. Headache prevalence following recovery from TTP and aHUS. Ann Hematol 2015;94:1473–6. https://doi.org/10.1007/s00277-015-2411-2.

[16] Yang M, Rendas-Baum R, Varon SF, Kosinski M. Validation of the Headache Impact Test (HIT-6^{TM}) across episodic and chronic migraine. Cephalalgia 2011;31:357–67. https://doi.org/10.1177/0333102410379890.

[17] Falter T, Böschen S, Schepers M, Beutel M, Lackner K, Scharrer I, et al. Influence of Personality, Resilience and Life Conditions on Depression and Anxiety in 104 Patients Having Survived Acute Autoimmune Thrombotic Thrombocytopenic Purpura. JCM 2021;10:365. https://doi.org/10.3390/jcm10020365.

[18] Husson O, de Rooij BH, Kieffer J, Oerlemans S, Mols F, Aaronson NK, et al. The EORTC QLQ-C30 Summary Score as Prognostic Factor for Survival of Patients with Cancer in the "Real-World": Results from the Population-Based PROFILES Registry. The Oncologist 2020;25:e722–32. https://doi.org/10.1634/theoncologist.2019-0348.

[19] Schwarz R, Hinz A. Reference data for the quality of life questionnaire EORTC QLQ-C30 in the general German population. European Journal of Cancer 2001;37:1345–51. https://doi.org/10.1016/S0959-8049(00)00447-0.

[20] Aaronson NK, Ahmedzai S, Bergman B, Bullinger M, Cull A, Duez NJ, et al. The European Organization for Research and Treatment of Cancer QLQ-C30: A Quality-of-Life Instrument for Use in International Clinical Trials in Oncology. JNCI Journal of the National Cancer Institute 1993;85:365–76. https://doi.org/10.1093/jnci/85.5.365.

[21] Page EE, Jiang Y, Terrell DR, Vesely SK, George JN. Long-term outcomes of health-related quality of life following diverse thrombotic microangiopathy syndromes: HRQOL following recovery from TMA. Am J Hematol 2016;91:E278–9. https://doi.org/10.1002/ajh.24315.

[22] Deford CC, Reese JA, Schwartz LH, Perdue JJ, Kremer Hovinga JA, Lämmle B, et al. Multiple major morbidities and increased mortality during long-term follow-up after recovery from thrombotic thrombocytopenic purpura. Blood 2013;122:2023–9. https://doi.org/10.1182/blood-2013-04-496752.

[23] Kroenke K, Spitzer RL. Patient Health Questionnaire-8 2018. https://doi.org/10.1037/t64702-000.

[24] Strine TW, Kroenke K, Dhingra S, Balluz LS, Gonzalez O, Berry JT, et al. The Associations Between Depression, Health-Related Quality of Life, Social Support, Life Satisfaction, and Disability in Community-Dwelling US Adults. Journal of Nervous & Mental Disease 2009;197:61–4. https://doi.org/10.1097/NMD.0b013e3181924ad8.

[25] Kroenke K, Strine TW, Spitzer RL, Williams JBW, Berry JT, Mokdad AH. The PHQ-8 as a measure of current depression in the general population. Journal of Affective Disorders 2009;114:163–73. https://doi.org/10.1016/j.jad.2008.06.026.

[26] Beck AT, Steer RA, Carbin MG. Psychometric properties of the Beck Depression Inventory: Twenty-five years of evaluation. Clinical Psychology Review 1988;8:77–100. https://doi.org/10.1016/0272-7358(88)90050-5.

[27] Chaturvedi S, Oluwole O, Cataland S, McCrae KR. Post-traumatic stress disorder and depression in survivors of thrombotic thrombocytopenic purpura. Thrombosis Research 2017;151:51–6. https://doi.org/10.1016/j.thromres.2017.01.003.

[28] Terrell DR, Tolma EL, Stewart LM, Shirley EA. Thrombotic thrombocytopenic purpura patients' attitudes toward depression management: A qualitative study. Health Sci Rep 2019;2. https://doi.org/10.1002/hsr2.136.

[29] Kroenke K, Spitzer RL, Williams JBW. The PHQ-9: Validity of a brief depression severity measure. J Gen Intern Med 2001;16:606–13. https://doi.org/10.1046/j.1525-1497.2001.016009606.x.

[30] Falter T, Schmitt V, Herold S, Weyer V, von Auer C, Wagner S, et al. Depression and cognitive deficits as long-term consequences of thrombotic thrombocytopenic purpura: DEPRESSION AND COGNITIVE DEFICITS IN TTP. EDITORIAL 2017;57:1152–62. https://doi.org/10.1111/trf.14060.

[31] John Rush A, Giles DE, Schlesser MA, Fulton CL, Weissenburger J, Burns C. The inventory for depressive symptomatology (IDS): Preliminary findings. Psychiatry Research 1986;18:65–87. https://doi.org/10.1016/0165-1781(86)90060-0.

[32] Rush AJ, Gullion CM, Basco MR, Jarrett RB, Trivedi MH. The Inventory of Depressive Symptomatology (IDS): psychometric properties. Psychol Med 1996;26:477–86. https://doi.org/10.1017/S0033291700035558.

[33] Zigmond AS, Snaith RP. The Hospital Anxiety and Depression Scale. Acta Psychiatr Scand 1983;67:361–70. https://doi.org/10.1111/j.1600-0447.1983.tb09716.x.

[34] Lovibond PF, Lovibond SH. The structure of negative emotional states: Comparison of the Depression Anxiety Stress Scales (DASS) with the Beck Depression and Anxiety Inventories. Behaviour Research and Therapy 1995;33:335–43. https://doi.org/10.1016/0005-7967(94)00075-U.

[35] Blevins CA, Weathers FW, Davis MT, Witte TK, Domino JL. The Posttraumatic Stress Disorder Checklist for *DSM-5* (PCL-5): Development and Initial Psychometric Evaluation: Posttraumatic Stress Disorder Checklist for *DSM-5*. JOURNAL OF TRAUMATIC STRESS 2015;28:489–98. https://doi.org/10.1002/jts.22059.

[36] Spitzer RL, Kroenke K, Williams JBW, Löwe B. A Brief Measure for Assessing Generalized Anxiety Disorder: The GAD-7. Arch Intern Med 2006;166:1092. https://doi.org/10.1001/archinte.166.10.1092.

[37] Wagnild GM, Young HM. Development and psychometric evaluation of the Resilience Scale. J Nurs Meas 1993;1:165–78.

[38] Scheier MF, Carver CS, Bridges MW. Distinguishing optimism from neuroticism (and trait anxiety, self-mastery, and self-esteem): A reevaluation of the Life Orientation Test. Journal of Personality and Social Psychology 1994;67:1063–78. https://doi.org/10.1037/0022-3514.67.6.1063.

[39] Beblo T, Kunz M, Brokate B, Scheurich A, Weber B, Albert A, et al. Entwicklung eines Fragebogens zur subjektiven Einschätzung der geistigen Leistungsfähigkeit (FLei) bei Patienten mit psychischen Störungen. Zeitschrift für Neuropsychologie 2010;21:143–51. https://doi.org/10.1024/1016-264X/a000013.

[40] Cella D, Riley W, Stone A, Rothrock N, Reeve B, Yount S, et al. The Patient-Reported Outcomes Measurement Information System (PROMIS) developed and tested its first wave of adult self-reported health outcome item banks: 2005–2008. Journal of Clinical Epidemiology 2010;63:1179–94. https://doi.org/10.1016/j.jclinepi.2010.04.011.

[41] Reilly MC, Zbrozek AS, Dukes EM. The Validity and Reproducibility of a Work Productivity and Activity Impairment Instrument: PharmacoEconomics 1993;4:353–65. https://doi.org/10.2165/00019053-199304050-00006.
